# Influence of Calcified Canals Localization on the Accuracy of Guided Endodontic Therapy: A Case Series Study

**DOI:** 10.3390/dj11080183

**Published:** 2023-07-28

**Authors:** Emanuele Ambu, Benedetta Gori, Crystal Marruganti, Giulia Malvicini, Antonietta Bordone, Lorenzo Giberti, Simone Grandini, Carlo Gaeta

**Affiliations:** 1 Unit of Endodontics and Restorative Dentistry, Department of Medical Biotechnologies, University of Siena, 53100 Siena, Italygiulia.malvicini@student.unisi.it (G.M.);; 2 Private Practice in Marsiglia, 13008 Marseille, France; 3 Dental Technician in Bologna, 40129 Bologna, Italy

**Keywords:** access cavity, CBCT, endodontics, guided endodontics, pulp canal obliteration

## Abstract

This study aimed to evaluate the precision of the guided endodontic technique applied to calcified canals in anterior teeth in relation to demographic and dental variables. The present observational study was conducted during the period 2020–2021. The patients were consecutive referrals at the Department of Endodontics and Conservative Dentistry of the University Hospital of Siena. The guided endodontics protocol was applied using 0.75 mm diameter burs for the lower teeth and 0.90 mm for the upper teeth. The inclusion criteria were as follows: (i) teeth with pulp canal obliteration (PCO) associated with a periapical lesion (periapical index (PAI) ≥ 2); (ii) teeth with PCO that require the placement of a root canal post for the execution of a prosthetic treatment; (iii) teeth in which surgical treatment was not justified. Socio-demographic characteristics of the patients were recorded and related to the drill path accuracy through the guide in the calcified endodontic canal, evaluated through a radiographic analysis, and classified as optimal (in the center of the root canal) and acceptable (deviated peripherally/tangentially). A logistic regression model was built to predict the factors that influence the poor precision of the technique. Seventeen patients (mean age 48 years) with eighteen calcified single-rooted teeth were enrolled. All teeth were associated with periapical lesions with PAI scores from 2 to 5 (mean PAI: 3.055). From the model, it is evident that the presence of a calcification affecting the apical area of the root increases the probability of being off-center with the bur by about 15 times. In addition, a previous attempt at endodontic treatment and the position in the lower arch increases the probability of non-centrality of the drill, although in a non-statistically significant way. In any of the analyzed cases, the guided endodontic technique applied to PCO did not determine the presence of iatrogenic errors, such as perforations. However, the apical localization of the obliteration increases the probability of being off-center with the drill during the instrumentation phase by about 15 times.

## 1. Introduction

Calcification of the root canal, also called pulp canal obliteration (PCO), is a pathological condition that often follows concussive or subluxation trauma [1], which determines an acceleration of dentin deposition [1]. It is characterized by hard tissue accumulation inside the canal and yellow color of the crown due to dentin increase with loss of coronal translucency [2]. A previous study, conducted on 122 teeth between 10 and 22 years after the trauma, showed complete PCO in 78 elements (63.9%) and partial PCO in the remaining 44 elements (36%) [3]. Indications for endodontic treatment are the presence of pain and periapical lesions. Pulp necrosis is not always present, and it can occur after several years in over 38% of cases, with 27.1% of these elements presenting pain on percussion, swelling, and/or a sinus tract, as well as a PAI ≥ 3 [4].

The American Association of Endodontics [5] considers the treatment of PCO as a high-complexity treatment even when performed with an operating microscope [6]. The risks associated with the traditional treatment of PCO are an over-extension of the coronal access with large tissue removal, iatrogenic perforations, non-retrieval of the root canal system, instrument fracture, and loss of the original canal path [7]. All the above-mentioned conditions may prevent the clinician from reaching the working length [2].

In the last 15 years, the use of customized 3D guides has been proposed for some endodontic treatments [8,9]. The use of a guide, programmed by superimposing the patient’s Digital Imaging and Communications in Medicine (DICOM) data with the Standard Triangle Language (STL) data derived from the optical impression, was mediated with the help of digital ones [10] already employed in implantology [7].

Periapical surgery was the initial indication for endodontic guides [11]. More recently, the guided technique has also been used to remove fiber posts and materials inside the canals [12]. However, one of the most successful indications is PCO treatment as an alternative to conventional access cavity preparation [10]. This approach consists in the use of a guide that enables the clinician to gain direct access to the patent part of the canal [13] with a bur of a variable diameter ranging between 0.75 mm and 1.2 mm to minimize the risk of procedural errors [7]. Furthermore, the correct use of this technique could avoid root perforations and allow treatment of these cases without a microscope [9]. Another ex vivo study demonstrated even greater accuracy, showing how microguided endodontics offers a fast and operator-independent technique [14]. Indeed, the digitally guided approach could help inexperienced clinicians to treat complex cases without a microscope [13,15].

However, despite the high level of success, there is a lack of evidence on the safe use and the possible limitations of this approach on specific patients, such as those with limited mouth opening or with aligners [16]. The aim of the current study was to investigate possible clinical variables which can predict the factors that influence the precision of the guided endodontic technique applied to calcified canals in anterior teeth.

## 2. Materials and Methods

This observational study was designed and realized according to the STrengthening the Reporting of Observational studies in Epidemiology (STROBE) statements when applicable. All consecutive patients attending the Unit of Endodontology and Restorative dentistry, School of Dentistry, University of Siena were screened in the period ranging from January 2020 to May 2021.

The inclusion criteria were as follows: (i) patients with PCO and a periapical lesion (PAI greater than or equal to 2), (ii) patients with PCO and dyschromia but without periapical lesion evident on intraoral X-ray (PAI less than 2), (iii) patients with PCO and the need for reconstruction with intraradicular retention, (iv) patients with PCO in whom a previous attempt to treat had failed, (v) patients able to provide written informed consent. Exclusion criteria: (i) teeth with calcification adjacent to teeth with metal restorations that would generate artifacts on CBCT, (ii) teeth with pathological mobility. The selected patients read and signed the written informed consent form in accordance with the Declaration of Helsinki.

### 2.1. Design and Execution of the Guide

All patients underwent a CBCT scan using the Hyperion X 5 (MyRay, Cefla, Imola, Italy). The selected FOV was the lowest available (6 × 6 cm in the upper jaw–6 × 7 cm in the lower jaw), using the advanced function (160-micron voxel) and then reconstructing the raw data with 80-micron voxels. Simultaneously, an optical impression was taken using an intraoral scanner (Aadva, IOS 100P, GC, Leuven, Belgium) to obtain a virtual model of the patient’s arch. The STL files derived from the intraoral scan were superimposed on the patient’s DICOM files using the RealGuide software (3diemme, Cantù, Italy). At least 3 common points to both volumes of data were taken to allow correct alignment, and then, where necessary, the appropriate corrections were made. Once the virtual model was obtained, virtual burs were superimposed, suitably created to reproduce burs of 22 mm in length and of 0.75 for the teeth of the mandible and 0.90 mm for the upper jaw, positioning the tip apically at the most coronal point where the calcification-free canal could be detected. On the other hand, the most coronal part of the virtual bur was passed through the palatal or lingual surface of the element to treat. A virtual sleeve was also designed to maintain the correct direction of the bur during use. The dimensions of the sleeve were the same as the real sleeve delivered by the manufacturer, together with the burs (external diameter of 3.5 mm, internal diameter of 0.75 or 0.90 mm, and height of 5 mm). The final generated model was then exported as an STL file and sent to a 3D printer (SprintRay Pro 95 DLP Technology, SprintRay, Los Angeles, CA, USA).

Once the guide is produced, it shows the most coronal part of the sleeve in a position that allows the tip of the bur, once inserted, to place itself (down to a depth of 22 mm) and to reach the most coronal point of the patent canal. In addition, openings are made on its surface as they are necessary to check that the guide is correctly positioned in the arch.

### 2.2. Clinical Procedure

#### 2.2.1. Teeth with Optimal Bur Course

**Figure 1:**
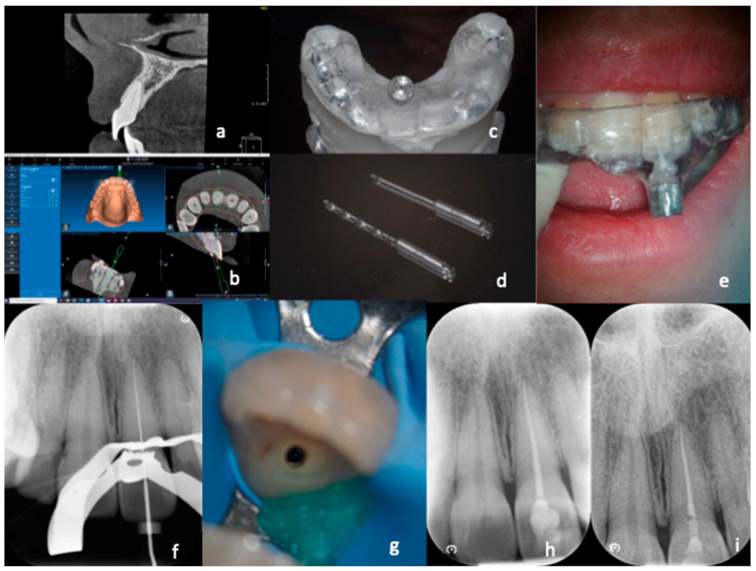
Treatment with “optimal precision” of a calcified canal. After carrying out a small FOV CBCT (**a**), which highlights the calcification of the canal, the DICOM data of the CBCT are coupled with the STL data derived from the digital impression (**b**). The result is a digital project that enables creation of a guide (**c**) which allows insertion of the two drills with a diameter of 0.9 mm up to the desired depth to reach the canal (**d**). The guide is inserted (**e**) and proceeds, with short excursion movements, to reach the canal. After rubber dam application, the working length is determined (**f**). Dentin removal is reduced even in the most coronal segment (**g**). Photos from the end of the treatment revealed correct obturation of the root canal system (**h**). A control X-ray was taken after six months (**i**).

During the appointment with the patient (Figure 1), the correct positioning and the stability of the template were tested, checking the tightness and the proper insertion thanks to the small windows opened in the occlusal part. None of the cases required template correction. At this point, local anesthesia was performed using articaine 1:100,000, and the template was inserted with no rubber dam in order to promote stability. A pencil tip was inserted into the sleeve (0.70 or 0.90 mm in diameter, depending on the size of the chosen bur), and the penetration point of the bur was marked on the enamel. The enamel was then removed with a round diamond bur mounted on a red handpiece until the dentin was reached. The template was removed, and the canal was washed with saline solution to cool down the tooth and remove debris. Again, the guide was positioned, and the dentin was removed, inserting the bur 2–3 mm using a blue handpiece at 10,000 rpm. Each maneuver was followed by removing the guide, washing with physiological solution, and checking the cavity with the operating microscope. Every three steps, the bur was removed from the handpiece and inserted into the canal to ensure its correct orientation. The guide was removed when the bur reached the maximum depth required to access the patent canal. After washing with physiological solution, a stainless-steel endodontic instrument was inserted (C+ File Ø 10, Dentsply Maillefer, Ballaigues, Switzerland CH), attempting to penetrate the canal. If this occurred, the canal was irrigated with 5.25% sodium hypochlorite after rubber dam isolation. Afterward, scouting of the canal was performed using a C+ File Ø 06 (Dentsply Maillefer, Ballaigues, Switzerland CH), connected with the apex locator (Root ZX, J. Morita Corporation, Tokyo, Japan), until WL was reached. The WL was immediately confirmed with a peri-apical X-ray. Coronal enlargement was carried out first with Scout RaCe (sizes 10, 15, and 20; taper 0.02) and then with Race series (sizes 20, 25, and 30; taper 0.04) instruments (FKG dentaire, La Chaux du Fonds, CH), while if the foramen was larger than 30, greater-diameter instruments were chosen. Each instrument insertion was followed by a wash with 1 cc of 5.25% sodium hypochlorite. After final cleansing with 5.25% sodium hypochlorite and 17% EDTA, the canal was rinsed with physiological solution, dried with absorbent paper cones, and sealed with bioceramic cement sealer (Bioroot RCS, Septodont, Saint Maur des Fosses, France) by using the single cone technique.

#### 2.2.2. Teeth with Acceptable Bur Course

**Figure 2:**
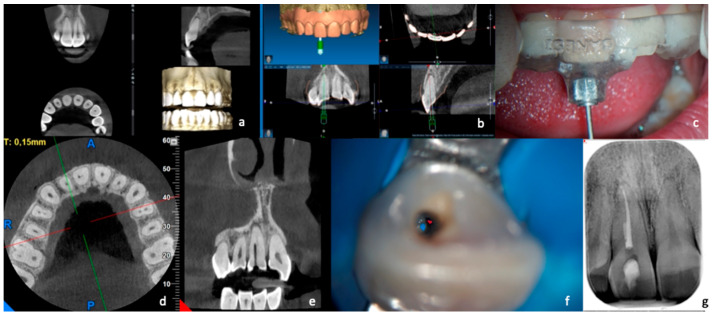
Treatment with “acceptable precision” of a calcified canal. The CBCT scan (**a**) and the optical impression were performed in this case. First, the data were processed to provide a virtual design of the guide (**b**), and then the artifact was created that was placed in position in the patient’s arch (**c**). Next, the drill was inserted up to the end of the “limit stop” and then, after several attempts, not finding the canal, a CBCT (**d**,**e**) was performed, which allowed highlighting of the position of the maximum penetration of the drill with respect to the patent canal. Finally, with the help of the microscope and ultrasound, the canal was found (**f**), shaped, and obturated (**g**).

For what regards those elements in which the canal was not immediately reached after the complete penetration of the bur (Figure 2), after several attempts made with the C+ steel instruments, two intraoral X-rays were carried out in different projections to locate the most apical point reached by the bur in relation to the beginning of the patent canal. In cases where it was impossible to reach the canal, a second CBCT examination according to a previous study [17] was carried out, with the same characteristics as the diagnostic one, requiring the patient to give written consent to this examination, as already stipulated in the first informed consent form.

The purpose was to understand where the tip of the bur ended up and the relationship of the patent channel with the point reached. For this purpose, the axial sections of the volumetric examination were primarily used. After the distance between the patent canal and the endpoint of the bur was recorded, the canal was widened using thin ultrasonic tips (ET 20-Satelec, Acteon, Merignac, France) in the direction of the point of canal patency under the control of the operating microscope (Pico-Zeiss, Oberkochen, Germany). Once access to the patent canal was detected, the endodontic treatment was carried out in the same way as indicated for the treatment of the canal with an optimal course.

### 2.3. Data Collection

Pre-, intra-, and post-operative data were collected to perform a statistical analysis to evaluate the precision of the bur penetration path.

#### 2.3.1. Pre-Operative Data

Information regarding patients’ age, sex, medical history (ASA, medications, and patient diseases), and dental history (history related to the etiology of the calcification) was registered. Moreover, data regarding the objective examination of the dental element showing calcification were registered. Precisely, information regarding the presence of prosthetic restorations, previous endodontic treatment attempts, conservative reconstructions, and fractures of the dental crown was recorded, as well as cold vitality test, pain on palpation, pain on percussion, periodontal probing, degree of mobility, and presence of discoloration. All participants received a periapical X-ray to determine the PAI [18]. Furthermore, DICOM data were obtained from CBCT to record: total length of the tooth and of the root, CEJ–obliteration distance, and degree of root canal calcification expressed as pulp space obliteration (PSO). The PSO was computed as CEJ–obliteration/total root length.

#### 2.3.2. Intraoperative Data

Data regarding the size of the apical preparations were registered and intraoperative radiographic examinations were performed to record: length of the drill path (path of the drill in the obliterated canal), drill path relative to the root (calculated by CEJ), and accuracy of the drill path.

#### 2.3.3. Post-Operative Data

One week after surgery, post-operative data regarding the total time of the intervention and anti-inflammatory intake were recorded. Moreover, post-operative pain was evaluated using the 10 mm visual analogue scale (VAS) which was divided into 10 equal intervals from 0 (no pain) to 10 (very severe pain).

### 2.4. Statistical Analysis

The statistical analysis was conducted using ad hoc statistical software (STATA BE, version 17, StataCorp LP, Cuyahoga Falls, TX, USA), setting the significance level at α = 0.05.

#### 2.4.1. Descriptive Statistics

Data obtained from clinical and radiographic measurements were expressed as variables. Continuous variables were expressed as mean ± standard deviation with the confidence interval (95% CI); categorical and dichotomous variables were expressed as “*n*” referring to the number of observations and the relative proportion (%).

#### 2.4.2. Inferential Statistics

The normality of the results was analyzed by Shapiro–Wilk test. Afterward, the parametric *t*-Student test was used for independent samples for the continuous variables. On the contrary, a chi^2^ test was carried out for categorical variables to perform subgroup analysis according to different characteristics:

- Localization of the calcification in the coronal or apical third. The ratio CEJ–obliteration/total length of the root was calculated. This ratio gave a value equal to 48%; if CEJ–obliteration ≤ 48% of the root length, the calcification is coronal, while if CEJ–obliteration > 48% of the root, the calcification is apical.
- Distance between the drill path and the obliteration (DPPSO), where the drill path (DP) is the drilling path of the bur with respect to the CEJ and the obliteration is calculated concerning the CEJ; in the analysis, DPPSO is mentioned, considering when the DP is greater or less than the PSO.
- Localization of the element in the maxillary or mandibular teeth (position of the calcified dental elements at the level of the upper or lower arch).

### 2.5. Logistic Regression Model

A logistic regression model was then designed for predictive purposes. The drill path accuracy (yes/no) was taken as model response variable. A chunk test was performed to choose the best model using the following independent variables: binary PSO (calcification present in the apical third of the canal), previous endodontic treatment (present), position of the dental element (lower arch), age, gender.

The best model was chosen based on the highest value of the area under the curve (AUC) and the lowest values of AIC and BIC. The regression model was statistically significant considering the likelihood ratio when *p* < 0.05.

## 3. Results

Seventeen patients with a total of eighteen calcified anterior teeth were included in the present study. During the clinical examination, none of the cases showed a positive response to the cold sensitivity test (Endo Ice Refrigerant Spray, Coltene; Lakeway, OH, USA). Ten patients were positive on palpation and five on percussion. All the considered dental elements presented physiological periodontal probing and mobility. In none of the cases did significant perforations nor deviations occur along the path of the drill with respect to the long axis of the treated tooth, and no patient needed to take pain-relieving drugs in the week following treatment (VAS = 0).

Table 1a,b show the clinical and radiographic variables of the dental elements included in the research. Table 1a indicates continuous variables, while Table 1b indicates binary variables. Inferential statistics for binary PSO, DPPSO and tooth position are shown in Table 2, Table 3 and Table 4.

In the logistic regression model, the response variable is binary (Table 5). Precisely, the value 0 indicates that the drill is centered while value 1 indicates that the drill is not centered. Therefore, this model aims to predict the factors that influence the probability of non-centered bur insertion. The independent variables included in the best model are binary PSO, previous endodontic treatment of the canal, and position of the dental element, with the values of AUC = 799, AIC = 21.7, and BIC = 24.3. The response variable considered is the offset of the drill (accuracy = 1). From this, it follows that when the exposure factor is binary PSO, the model indicates that the calcification in the most apical position increases the probability of the drill being decentered by about 15 times. This value is statistically significant (*p*-value = 0.046).

Moreover, the probability of being off-center increases with a previous endodontic treatment attempt and with the tooth’s position in the lower arch, but these values do not show statistical significance. The model can interpret response variables equal to 23% and highlights how the presence at baseline of a calcification at the apical level increases the probability of being off-center with the drill by about 15 times.

## 4. Discussion

Calcification of the root canal system is a condition that requires complex treatment, both for general dentists and endodontic specialists. It is a very time-consuming therapy with a high risk of dentin removal and an increased risk of perforation [10]. PCO often occurs after trauma [19], caries, chamber pulpotomies [20], conservative or orthodontic therapies [21], or, physiologically, by the apposition of dentin during life in elderly patients [22]. The use of the digitally guided technique in the treatment of PCO is relatively recent, and the literature has not yet thoroughly examined its strength and limitations.

The aim of the present study was to evaluate the precision of the guided endodontic technique applied to calcified canals in anterior teeth. The regression model demonstrated how an apically extended calcification increases the probability of being off-center with the bur by about 15 times, with a statistically significant value (*p*-value = 0.046). Furthermore, a previous endodontic treatment increases the odds of non-centrality of the drill, although in a non-statistically significant way.

In the present study, a volumetric CBCT examination with a limited FOV and slices smaller than one hundred microns were used in accordance with a recently published article, which demonstrated that the exam should be performed with the “lowest possible” FOV and, ideally, with the highest resolution, to best highlight the residual canal present in the root [10]. In other works, such as that of Zehnder and colleagues, CBCT with 0.125-micron slices has been used [9].

Regarding accuracy, one of the first works on the guided technique demonstrated an average deviation of 0.46 mm between the target point and the center of the drill path of the bur [8]. Another ex vivo study [9] highlighted the accuracy, with a mean deviation of the drill tip of 0.17 mm in the coronal–apical direction, 0.27 in the mesio-distal direction, 0.47 in the oral–buccal direction, and an average deviation angle of 1.81°. The same authors conducted an ulterior ex vivo study [14] using a smaller drill (0.85 mm), obtaining a higher accuracy. Those results showed an average deviation in the mesio-distal direction of 0.14 mm, 0.34 in the bucco-oral direction, 0.12 in the coronal–apical direction, and an average angle of deviation of 1.59°. Recently, another ex vivo study [23] was carried out on 84 extracted teeth, including incisors, premolars, and molars, for 117 canals, of which 23 were recorded as inaccessible. After the guided opening, all the canals were practicable, and the deviations for all groups were 0.13 ± 0.21 mm at the coronal position, 0.46 ± 0.4 mm at the apical position, and 2.8° ± 2.6° in angular deviation. The most considerable deviations, with statistically significant differences, were found for the molars.

A recent in vivo study [7] analyzed the accuracy of the technique in 50 consecutive cases. In this work, the teeth were divided into two groups based on the relationship between the drill path and the length of canal obliteration (distance between CEJ and the apical end of the obliteration), with a median value of 43%. The precision of the glide path was then evaluated concerning the length of the obliteration, creating two groups, namely “optimal precision” where the glide path was centered and “acceptable precision” where the glide path was shifted peripherally or tangentially. In most cases (28/50), accuracy was “acceptable” while, in 22/50, it was optimal. The canal search was carried out by progressively inserting the drill and checking with X-rays and the operating microscope for the reaching of the patent canal. In cases where this was not possible when the maximum depth of the drill was reached, the guide was removed, and a further attempt was made using the space already prepared as a guide. Moreover, if this step was unsuccessful, a second CBCT was performed to verify the position of the canal with respect to the point reached by the drill tip. The canal was reached after gently removing the dentin peripherally using the operating microscope and ultrasonic inserts. According to the literature, given the two-dimensional nature of the radiographic images, the deviation of the access cavity could be underestimated in the bucco-lingual projection [7].

In all treated cases, it was possible to reach the patent canal and undertake endodontic treatment without complications, such as perforations or large dentin removals. In the present study, the ratio between CEJ–obliteration and the total length of the root was calculated, with a value equal to 48%. This allowed the subdivision of a coronal group and an apical group. The distance between the drill path and the obliteration was then considered and calculated based on their distance from the CEJ. The elements were prepared using smaller-diameter burs than in other studies [7,8,9], using 0.90 mm burs for the upper anterior elements and 0.75 mm for the anterior mandibular ones. In the literature, there is scarce information regarding the relationship between the dimension of the drill and a greater or lesser ability to remain centered in the glide path. Comparing the accuracy from previous studies [9,14], a better result arises when a 0.85 mm diameter bur is used compared to the 1.125 mm one. Moreover, the drill speed used in the current study was 10,000 rpm, which is much higher compared to the one used in other studies [7,8]. However, the influence of the drill speed on the maintenance of the drill’s centering with respect to the open part of the canal has not yet been investigated. It was recently demonstrated [7], analyzing the depth of the canal obliteration with regard to the precision of the drill path, that the calcification is deeper in the maxillary than in the mandibular teeth. This resulted in a more significant amount of “optimal penetrations” in maxillary teeth. However, in this previous study, no differences in precision were found based on the length of drill penetration and extent of calcification. Furthermore, the regression tests they performed did not provide additional information with respect to that already obtained with the chi^2^ test.

The purpose of the logistic regression model was to understand the factors that predict whether the drill is uncentered. The independent variables included in the model were binary PSO, previous endodontic treatment, and the position of the tooth in the arch (upper or lower). On the other hand, the presence of a previous treatment attempt and the tooth being in the mandibular arch increase the possibility of non-centered bur insertion, but in a non-statistically significant way (Table 4). The presence of an apically extended calcification appears to increase the odds of non-centered bur insertion by about 15 times with a value that is statistically significant (*p*-value = 0.046). From a clinical point of view, the indication that can be derived from the results is evident. If the calcification is in the most coronal half of the canal, it can be easily treated with the guided technique. This approach avoids excessive dentin removal and risks of root perforation that are otherwise present with the traditional technique [6]. If, on the other hand, the perforation reaches the apical half of the canal, the struggle in finding the canal increases by 15 times, suggesting that greater caution should be taken. Even if no root perforations were detected in the treated cases, recovering a deep but narrow canal (0.75–1.2 mm) can be extremely difficult, mainly if an operating microscope and suitable ultrasonic inserts are not used. The positioning of the canal with respect to the point reached by the drill may also require the use of several intraoral radiographs or an additional CBCT examination. Overall, only clinicians who have access to and the ability to use tools such as an operating microscope and CBCT should perform the treatment of the most apical cases.

However, despite the high level of accuracy and success, guided endodontics has some disadvantages, such as the increased time required for treatment planning and higher costs for the patients compared to the traditional approach [10].

Some limitations must be considered such as the small number of samples, the absence of remote patient monitoring, and the lack of a comparison between the guided and traditional techniques.

## 5. Conclusions

The correct use of the guided endodontic technique could avoid root perforations and allow treatment of these cases without a microscope, however, to improve the evidence for guided endodontics high-quality clinical studies as required.

The guided endodontic technique applied to PCO did not cause iatrogenic errors, such as perforations, in any of the analyzed cases. However, the presence of a calcification affecting the apical third increases the odds of being off-center with the bur by about 15 times. In addition, there are two additional causes that could influence the probability of non-centered bur insertion: a previous attempt at endodontic treatment and the tooth positioned in the lower arch. However, there was no statistically significant difference related to those factors regarding bur orientation.

## Figures and Tables

**Table 1:** Descriptive statistics: clinical and radiographical variables.

**a.** Continuous variables.	
**Variables**	Mean (95% CI/n(%))
Age	48.36 ± 17.96 [39.43, 57.30]
Pulp Space obliteration	0.409 ± 0.141 [0.34, 0.48]
Drill path relative to the root	0.415 ± 0.1610 [0.34, 0.50]
Drill path relative to CEJ	5.05 ± 2.10 [4.01, 6.10]
Drill path length	10.87 ± 2.05 [9.86, 11.90]
Drill path accuracy	107.22 ± 170.11 [22.63, 191.82]
Root total length	12.65 ± 1.99 [11.66, 13.64]
CEJ–obliteration	5.29 ± 1.94 [4.32, 6.28]
Time	53.88 ± 11.57 [48.13, 59.65]
PAI score	3.05 ± 1.10 [2.50, 3.60]
1-week VAS scale	0 ± 0 [0, 0]
**b.** Binary variables.	
**Variables**	n (%)
Tooth position	
Superior	14 (77.78%)
Inferior	4 (22.22%)
Endodontic treatment	
Yes	14 (77.78%)
No	4 (22.22%)
Apical diameter	
25	1 (5.56%)
30	16 (88.89%)
50	1 (5.56%)
Taper	
0.04	18 (100%)
Drill path accuracy	
Off-center	7 (38.89%)
Centered	11 (61.11%)
Anti-inflammatory drugs	
Yes	13 (72.22%)
No	5 (27.78%)
Binary PSO ^1^	
Coronal	13 (72.22%)
Apical	5 (27.78%)
DPPSO ^2^	
DP ≤ PSO	12 (66.67%)
DP > PSO	6 (33.33%)

^1^ Binary pulp space obliteration (PSO) = cementoenamel junction (CEJ–obliteration/total length of the root ratio. This ratio gave a value equal to 48%. If CEJ–obliteration ≤ 48% of the root length, then the calcification is coronal, while if CEJ–obliteration > 48% of the root, the calcification is apical. DP = drill path related to CEJ; ^2^ DPPSO = distance between the drill path and the obliteration.

**Table 2:** Inferential Statistics for Binary Pulp Space Obliteration (PSO) (coronal vs. apical).

Variables	Mean (95% CI/n(%))		*p*-Value
	*Coronal*	*Apical*	
**AGE ^1^**	50.23 ± 19.46 [38.4, 61.9]	43.5 ± 13.95 [26.1, 60.8]	0.49
**Drill path accuracy ^2^**			0.047
**Centered**	10 (90.91%)	1 (9.09%)	
**Decentered**	3 (42.86%)	4 (57.14%)	
**Time ^1^**	52.69 ± 10.72 [46.2, 59.1]	57 ± 14.4 [39.1, 74.8]	0.49
**Drill path accuracy ^1^**	65.38 ± 128.1 [−12, 142.7]	216 ± 230.8 [−70.6, 502.6]	0.092
**Tooth position ^2^**			0.999
**Superior**	10 (71.43%)	4 (28.57%)	
**Inferior**	3 (75%)	1 (25%)	
**DPPSO ^2^**			0.114
**DP ≤ PSO**	7 (58.33%)	5 (41.67%)	
**DP > PSO**	6 (100%)	0 (0%)	

^1^ Binary PSO = cementoenamel junction (CEJ)–obliteration/total length of the root ratio. This ratio gave a value equal to 48%. If CEJ–obliteration ≤ 48% of the root length, then the calcification is coronal, while if CEJ–obliteration > 48% of the root, the calcification is apical. ^2^ DP = drill path related to CEJ.

**Table 3:** Inferential statistics for drill path and Obliteration distance (DPPSO) considering a drill path (DP) smaller or greater than the pulp space obliteration (PSO) (DP ≤ PSO vs. DP > PSO).

Variables	Mean (95% CI/n(%))		*p*-Value
	DP ≤ PSO	DP > PSO	
**Age ^1^**	42.48 ± 13.36 [33.9, 50.9]	60.13 ± 21.33 [37.7, 82.5]	**0.045**
**Drill path accuracy ^2^**			0.31
**Centered**	6 (54.55%)	5 (45.45%)	
**Decentered**	6 (85.71%)	1 (14.29%)	
**Time ^1^**	55 ± 12.24 [47.2, 62.7]	51.66 ± 10.8 [40.3, 63]	0.58
**Drill path accuracy ^1^**	135.83 ± 187.68 [16.5, 255]	50 ± 122.4 [−78.5, 178.5]	0.32
**Tooth position ^2^**			0.56
**Superior**	10 (71.43%)	4 (28.57%)	
**Inferior**	2 (50%)	2 (50%)	

^1^ *T*-Student test for continuous variables; ^2^ chi^2^ test for categorical variables

**Table 4:** Inferential statistics for Tooth Position (Maxillary vs. Mandibular).

Variables	Mean ± Standard Deviation [95% CI/n(%_)_]		*p*-Value
	Maxillary	Mandibular	
**Age ^1^**	43.22 ± 16.16 [33.8, 52.5]	66.36 ± 11.88 [47.4, 85.2]	**0.017**
**Drill path accuracy ^2^**			0.999
**Centered**	9 (81.82%)	2 (18.18%)	
**Decentered**	5 (71.43%)	2 (28.57%)	
**Time ^1^**	51.78 ± 8.9 [46.6, 56.9]	61.25 ± 17.96 [32.6, 89.8]	0.15
**Drill path accuracy ^1^**	95 ± 173 [−5.3, 195.3]	150 ± 173.2 [−125.6, 425.6]	0.58
**Binary PSO ^2^**			0.999
**Coronal**	10 (76.92%)	3 (23.08%)	
**Apical**	4 (80%)	1 (20%)	

^1^ *T*-Student test for continuous variables; ^2^ chi^2^ test for categorical variables.

**Table 5:** Logistic regression model with predictive purpose.

Best model (AUC = 0.799, AIC = 21.7, BIC = 24.3)						
**LR chi^2^**	**Prob > chi^2^**	**Pseudo R^2^**				
**5.50**	0.1384	0.2288				
**Drill path accuracy**	**OR**	**SE**	**z**	***p*-value**	**95% CI**	
					**Lower**	**Upper**
**Binary PSO**	15.55	21.42	1.99	**0.046**	1.044	231.56
**Endodontic treatment**	1.08	1.12	0.17	0.864	0.046	13.25
**Tooth position**	2.30	3.24	0.59	0.554	0.145	36.39
**Cons**	0.251	0.222	−1.56	0.119	0.4428	1.429

## Data Availability

The data presented in this study are available on request from the corresponding author.
